# PRO-MINE: A Bioinformatics Repository and Analytical Tool for *TARDBP* Mutations

**DOI:** 10.1002/humu.21393

**Published:** 2011-01

**Authors:** Sofia Pinto, Kristian Vlahoviček, Emanuele Buratti

**Affiliations:** 1 Bioinformatics Group, Department of Molecular Biology, Division of Biology, Faculty of Science, University of ZagrebCroatia, USA; 2 International Centre for Genetic Engineering and Biotechnology (ICGEB)Trieste, Italy

**Keywords:** Neurodegeneration, TDP-43, TDP43, TARDBP, Alzheimer, Amyotrophic Lateral Sclerosis, ALS, Fronto Temporal Lobar Degeneration, database

## Abstract

TDP-43 is a multifunctional RNA-binding protein found to be a major protein component of intracellular inclusions found in neurodegenerative disorders such as Fronto Temporal Lobar Degeneration, Amyotrophic Lateral Sclerosis, and Alzheimer Disease. PRO-MINE (PROtein Mutations In NEurodegeneration) is a database populated with manually curated data from the literature regarding all *TDP-43/TDP43/TARDBP* gene disease-associated mutations identified to date. A web server interface has been developed to query the database and to provide tools for the analysis of already reported or novel TDP-43 gene mutations. As is usually the case with genetic association studies, assessing the potential impact of identified mutations is of crucial importance, and in order to avoid prediction biases it is essential to compare the prediction results. However, in most cases mutations have to be submitted separately to various prediction tools and the individual results manually merged together afterwards. The implemented web server aims to overcome the problem by providing simultaneous access to several prediction tools and by displaying the results into a single output. Furthermore, the results are displayed together in a comprehensive output for a more convenient analysis and are enriched with additional information about mutations. In addition, our web server can also display the mutation(s) of interest within an alignment of annotated TDP-43 protein sequences from different vertebrate species. In this way, the degree of sequence conservation where the mutation(s) occur can be easily tracked and visualized. The web server is freely available to researchers and can be accessed at http://bioinfo.hr/pro-mine. © 2010 Wiley-Liss, Inc.

## INTRODUCTION

Genetic association studies aim to elucidate how single nucleotide polymorphisms (SNPs) may be related to a genetic predisposition to develop disease. Non-synonymous single nucleotide polymorphisms are a type of SNPs that result in a codon which codes for a different amino acid. Some of these polymorphisms are responsible for many diseases as they may lead to appreciable changes in protein structure and function. Annotating and distinguishing the polymorphisms that render the protein non-functional from those that do not is essential for the study of disease-associated mutations. However, screening the pathogenicity for each identified SNP experimentally is often a very laborious task that requires a considerable amount of time. To overcome this challenge, several computational approaches for *in silico* predictions have been developed in recent years, reviewed recently by Thusberg and Vihinen (Thusberg and Vihinen, 2009).

Nuclear factor TDP-43 (TDP43; HGNC-approved symbol TARDBP; MIM# 605078) is an example of a protein with high SNP count. TDP-43 is a multifunctional RNA binding protein that plays a role in several cellular processes such as transcription, pre-mRNA splicing, mRNA stability and mRNA transport (Buratti and Baralle, 2008; Buratti and Baralle, 2009; Chen-Plotkin et al., 2010). Recently, TDP-43 has also been found as the major protein component of the intracellular inclusions occurring in the neuronal tissues of patients affected by a series of neurodegenerative diseases which include Fronto Temporal Lobar Degeneration (FTLD-U), Amyotrophic Lateral Sclerosis (ALS) (Geser et al., 2009; Neumann et al., 2006) and in a significant proportion of Alzheimer Disease patients (Amador-Ortiz et al., 2007; Bigio, 2008; Rohn, 2008). In affected neurons, TDP-43 is abnormally mislocalized to the cytoplasm, ubiquitinated, hyperphosphorylated and cleaved to generate C-terminal fragments (Dormann et al., 2009; Hasegawa et al., 2008; Igaz et al., 2008; Neumann et al., 2009; Zhang et al., 2009). For a detailed clinical view of *TARDBP* mutation carriers in the aforementioned diseases the reader is referred to the Alzheimer Disease and Frontotemporal Dementia Mutation Database (http://www.molgen.ua.ac.be/ftdmutations) and to the Amyotrophic Lateral Sclerosis Online genetic Database (http://alsod.iop.kcl.ac.uk).

In the beginning, the question was still open regarding the relative importance of this protein: should TDP-43 protein inclusions be considered as a simple pathological curiosity of these diseases or did it really play a role in their origin and progression (Rothstein, 2007)? The recent development of several animal models, from simple organisms to more complex ones, has clearly shown the pathological potential of both TDP-43 depletion and overexpression in general (Feiguin et al., 2009; Hanson et al., 2010; Johnson et al., 2008; Kraemer et al., 2010; Li et al., 2010; Lu et al., 2009; Sephton et al., 2010; Wils et al., 2010; Wu et al., 2010).

**Figure 1:**
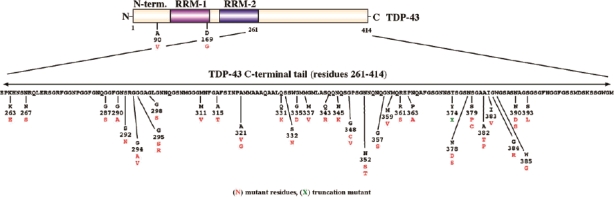
TDP-43 disease-associated mutations. The diagram shows the distribution of all missense mutations associated with disease along the TDP-43 protein: all mutations are reported in the C-terminal tail of the protein with the exception of p.Ala90Val and p.Asp169Gly (see *Database and Analysis Tools* section for details about the nomenclature used for mutations).

Another evidence that TDP-43 misregulation may be the cause of disease has been provided by several genetic findings that have identified TDP-43 mutations in about 5% of the patients, as recently reviewed by Pesiridis et al. (Pesiridis et al., 2009). With only two exceptions (p.Ala90Val and p.Asp169Gly) all disease-associated mutations in human TDP-43 are localized in the C-terminal tail of the protein (a region that in hnRNP proteins is usually associated with protein-protein interactions) and are all except p.Tyr374X characterized as missense mutations (Figure 1). In this respect, it is important to note that all these mutations are dominant traits and, until today, in the C-terminal tail no missense mutation has been found in healthy controls. At the moment, very little is known about the role potentially played by these mutations. Nonetheless, expression of mutant forms of this protein in *Mus musculus* (Wegorzewska et al., 2009), *Rattus norvegicus* (Zhou et al., 2010), *Danio rerio* (Kabashi et al., 2010), *Gallus gallus* (Sreedharan et al., 2008), *Saccharomyces cerevisiae* (Johnson et al., 2009) and neuronal cell lines (Barmada et al., 2010; Nonaka et al., 2009) can reproduce neurodegenerative effects similar to those observed in human disease.

Here we present PRO-MINE (PROtein Mutations In NEurodegeneration), a manually curated mutation repository providing detailed and updated information from the literature focusing on the potential functional consequences of the *TARDBP* gene mutations.

We designed and implemented a user-friendly web interface aimed to facilitate the access to the database and to integrate multiple prediction tools for the analysis of mutations. The results from the prediction tools are automatically retrieved into a single output, greatly reducing the amount of time required when studying the effects of mutations on protein. Furthermore, based on the fact that knowledge regarding the functional consequences of a mutation can also be gained from sequence comparison, PRO-MINE also offers the possibility to display an aligned set of protein sequences from several vertebrate species with the mutations of interest distinctly annotated.

PRO-MINE represents a novel approach and an advantage upon other existing tools and provides the proof of concept for the need to generally integrate data with computational analytical tools in the study of mutations.

## DATABASE AND ANALYSIS TOOLS

### PRO-MINE database

PRO-MINE is a relational database of published disease-associated mutations in human neurodegenerative diseases, in particular focusing on the *TARDBP* gene.

**Figure 2:**
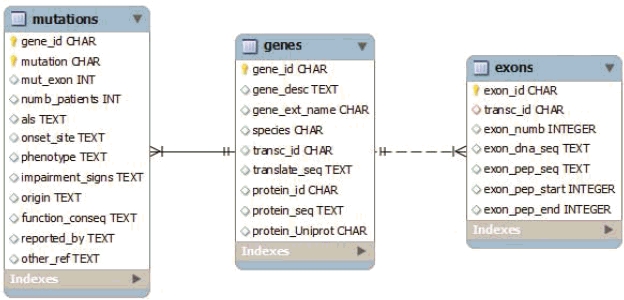
Entity relationship diagram of the PRO-MINE database. The scheme illustrates the tables created for data storage (*mutations, genes* and *exons*) with the respective attributes and how they are connected. The simple relationships between the tables facilitate the possible expansion of the database to other proteins involved in neurodegeneration.

The database was created using MySQL and consists of the following tables: *mutations, genes* and *exons*. The entity relationships of the three tables are depicted in Figure 2. The conceptual scheme described was designed to make easier future inclusion of other relevant genes and associated mutations. The database stores not only information related to the mutations identified in the human *TARDBP* gene but also protein and genomic data associated with the gene itself.

An exhaustive manual review of the literature and public repository databases regarding *TARDBP* gene mutations was performed. The result was a curated set of 43 mutations representing the most exhaustive to date status of identified and characterized TDP-43 gene mutations, further referred to as ‘TDP-43 mutations core set’. Relevant information concerning the mutations was, wherever available, extracted from the literature and included in the *mutations* table. The information provided about a given mutation for each entry of the mutations table includes: (1) the exon number where the mutation occurs; (2) clinical information related to the mutation such as the number of patients where it was identified (in case of mutations found in different affected family members only one patient for family was computed), the phenotype presented by the patients or the onset site of the disease; (3) a brief description of observed experimental evidences on the functional consequences of the mutation on a protein; and (4) references to original papers that reported the mutation as well as later studies. The mutation nomenclature complies with the accepted guidelines proposed by the Human Genome Variation Society (http://www.hgvs.org/mutnomen) and the variants numbering system is given on the protein level relative to the amino acid reference sequence.

Protein sequences and genomic data stored in the *genes* and *exons* tables were retrieved from Ensembl (release 57 – Mar 2010) (Hubbard et al., 2009) through its Perl Application Program Interface. The set of protein sequences comprises the human protein sequence encoded by the *TARDBP* gene (RefSeq NM_007375.3) and its orthologs from six vertebrates: *Pan troglodytes, Mus musculus, Rattus norvegicus, Gallus gallus, Xenopus tropicalis* and *Danio rerio*.

### PRO-MINE web server interface implementation

The PRO-MINE web server is intended to facilitate the access to the data stored in the database and to provide means for its analysis. The web server interface consists of dynamically generated HTML pages and was developed using Perl CGI scripts (http://www.perl.com). The scripting programs run on Apache web server and use MySQL (http://www.mysql.com) to retrieve and manage information stored in the database.

### Analysis Tools

The PRO-MINE web server integrates four available software tools – NetPhos 2.0 (Blom et al., 1999), PolyPhen (Ramensky et al., 2002), SIFT (Ng and Henikoff, 2003) and SNAP (Bromberg and Rost, 2007) – to predict the pathological character of a mutation on a protein. The prediction tools were obtained as stand-alone software packages (free for academic use) and integrated into the PRO-MINE web server with custom Perl scripts. The integration of the prediction tools serves the purpose of providing researchers with a layout for a more convenient and faster analysis of the results attained.

The four software tools use different models and features to predict the functional effect of mutations on protein. NetPhos uses sequence and structure-based neural networks for predicting phosphorylation sites at serine, threonine or tyrosine residues in protein sequences based on the premise that creation of new phosphoylation sites or disruption of existing ones can alter the functionality of a protein. Although in normal conditions *TARDBP* does not seem to be heavily phosphorylated, one of the distinguishing features of this disease-related protein is that it becomes phosphorylated at several residues in the C-terminal region (Hasegawa et al., 2008; Neumann et al., 2009). Therefore, prediction of phosphorylation sites should be taken into consideration because the aberrant phosphorylation in the C-terminal region of *TARDBP* may be very important with regards to altering its functionality. PolyPhen algorithm applies straightforward empirical rules to sequence-based, structural and phylogenetic parameters describing the mutation. SIFT is an evolutionary-based approach which relies solely on the information extracted from a multiple alignment of homologous proteins and the physical properties of amino acids to calculate the probability that a mutation will be tolerated. Finally, SNAP combines information derived from sequence homology and protein properties (secondary structure, solvent accessibility, etc) into neural networks. The reason for the use of complementary approaches lies with different strengths and weaknesses of various prediction algorithms, and in the absence of experimental evidence, the best strategy is to apply a consensus decision among as many prediction tools as possible.

Besides these analysis tools available for user selection, PRO-MINE uses the multiple alignment software for protein and nucleotide sequences – MUSCLE (Edgar, 2004) – in the internal pipeline. MUSCLE is employed in the protein sequence annotation analytical tool to perform on-the-fly multiple alignments of homologous protein sequences.

## DISCUSSION

Bioinformatics methods are of great help in elucidating the potential functional impact of mutations on protein since they provide quick and accurate predictions. Nonetheless, each of the methods applies different features and algorithms in the prediction analysis and, as a consequence, different results might arise. This poses a challenge for the choice of the best method to utilize. As intuitively obvious, the best strategy to overcome this problem is to use several prediction methods at the same time and to take into account all the results with the purpose of obtaining a more reliable evaluation. However, manual inspection and analysis of the results from several bioinformatics tools is a time-consuming procedure that requires parallel submission of the mutations of interest to each prediction server separately and manual combining of the resulting predictions afterwards. Usually, these tools require different input formatting and structure their output in different ways, and it is often impractical and time-consuming for the user to collect the data and integrate it in the proper context. Also, knowledge discovery process requires the researcher to use additional information sources and analysis tools.

In the PRO-MINE database and analysis server, the data and analysis tools are integrated in a user-friendly interface in order to shift the focus of the researcher towards the problem and away from manual tasks of data manipulation. Besides the manually curated database of known TDP-43 mutations, the analysis functions implemented in PRO-MINE include: (a) prediction of mutational effects on protein function and (b) protein sequence annotation with mutations. Details on the usage and currently available options are described below. The developed web tool is freely available at: http://bioinfo.hr/pro-mine.

### Input

**Figure 3:**
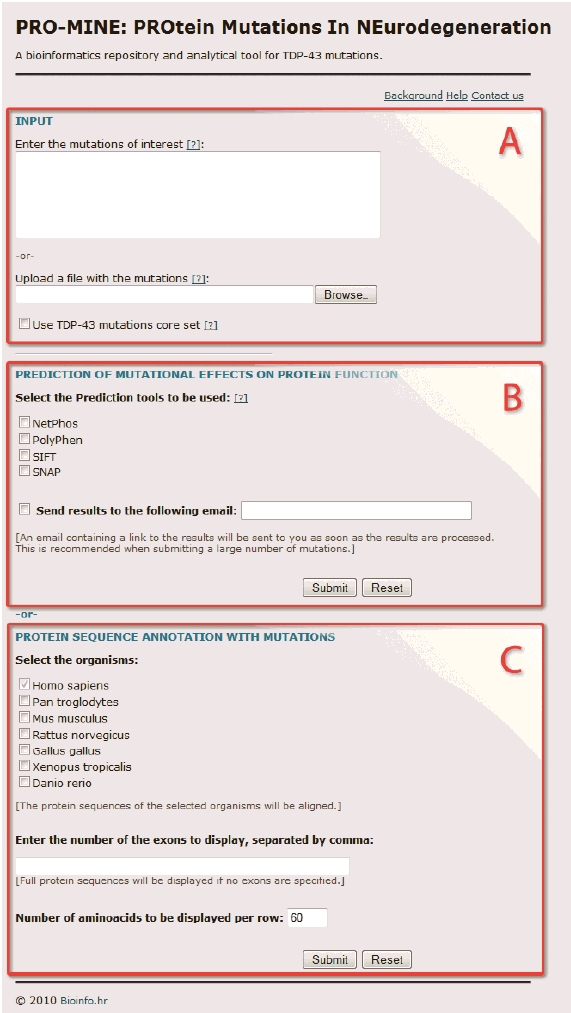
Screenshot of the PRO-MINE web server interface. **A.** Query input panel: mutations can be submitted using the text box or by uploading a file; the checkbox must be selected to use the TDP-43 mutations core set. **B.** Section to predict the potential effect of the entered mutations on protein function: the prediction tools can be chosen from the list shown; an e-mail address can be provided to receive the results (this is a recommended course of action since some of the predictions require a considerable amount of time to be processed). **C.** Section to display aligned protein sequences from different species with annotated query mutations: a list of the possible species to select is provided with the *Homo sapiens* option being permanently checked; it is also offered the possibility to align only amino acid sequences encoded by individual exons by specifying the exons numbers in the appropriate text box.

The web server accepts as input a list of mutations which can be typed directly into the query input box or uploaded as a simple text file (Figure 3a). Mutation nomenclature should follow the standard protein mutation format: *XposY*, where *X* and *Y* are the two amino acid variants (wild-type and mutant, respectively) and *pos* is the position of the substitution in the sequence (e.g., M337V). The server also offers the possibility of using the TDP-43 mutations core set by simply selecting the corresponding checkbox. Additionally, both fields can be used in combination – an option that can be especially useful to compare new mutations with previously identified ones.

### Prediction of mutational effects on protein function

By providing simultaneous access to several prediction tools, PRO-MINE renders the ability to obtain predictions more efficiently and in comparatively less time than manually combining analysis results from different sources. PRO-MINE integrates results from four separately selectable prediction tools (Figure 3b), previously described in the *Database and Analysis Tools* section.

**Figure 4:**
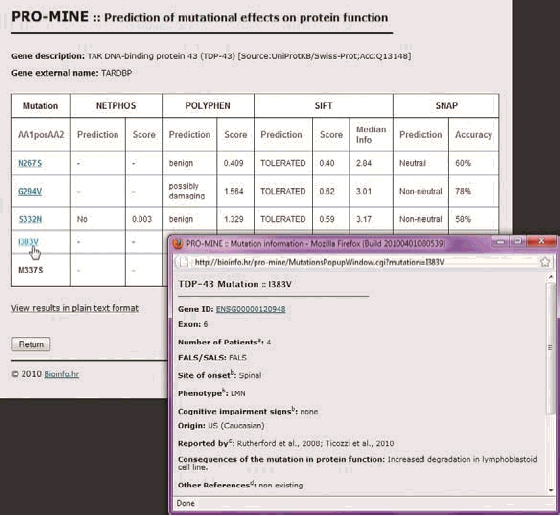
PRO-MINE output: prediction of mutational effects on protein function. In the table are displayed in a comprehensive manner the predictions for five submitted mutations given by NetPhos, PolyPhen, SIFT and SNAP. Each row of the table corresponds to the results of all selected web tools for a single mutation. Below the table there is a link to view or save the results in plain text format. Selecting a mutation identifier opens a pop-up window containing detailed data related to the mutation.

The results of the selected prediction tools are formatted and displayed in a single table, facilitating the comparison of the different predictions. Predictions regarding the TDP-43 mutations core set are pre-computed to avoid waiting time. Query results can also be viewed in plain text format or downloaded for further use. Additionally to the predictions, for the TDP-43 mutations core set, selecting the mutation identifier opens a new window displaying associated information such as clinical data about the patients presenting the mutation, a brief description of observed experimental effects of the selected mutation on protein function and the literature references reporting the mutation (see *Database and Analysis Tools*). An example of the results output for five mutations is shown in Figure 4.

It should be stressed out that the validity of *in silico* predictions should always be assessed by further experimental studies. For this reason, when known, observed experimental evidences on the functional consequences of the TDP-43 mutations core set were collected and can be easily accessed (as shown in the pop-up window in Figure 4).

The server offers two possibilities of use: the interactive use with results appearing in the web browser which will be the choice for most analyses as the computation time is relatively short; and the off-line use with e-mail report of the results which is recommended when a long list of mutations is submitted. In the latter case, the results will not appear directly on the screen but a link pointing to the results will be sent to the submitted e-mail address. A session identifier is assigned to each submitted job so that the results can be accessed at any time by following the link provided in the e-mail. Moreover, the link allows the results to be easily shared with other researchers.

### Protein sequence annotation with mutations

Multiple alignment of protein sequences from different species provides insight into the level of conservation of an amino acid in a sequence position. Conserved positions are commonly associated with functionally important sites and disease-related mutations frequently occur in those positions. Based on these considerations, an analytical tool that aligns (see *Database and Analysis Tools*) and displays the TDP-43 protein sequences encoded by the TDP-43 gene from different vertebrate species with the submitted mutations annotated was implemented.

The vertebrate species available include *Homo sapiens, Pan troglodytes, Mus musculus, Rattus norvegicus, Gallus gallus, Xenopus tropicalis* and *Danio rerio*. The *Homo sapiens* option is checked by default and cannot be unchecked while the other organisms are provided as choices (Figure 3c).

Alternatively to the complete protein sequence, the amino acid sequences encoded by the individual exons can be aligned and displayed separately, in case a more detailed analysis is required. In this case, the ordinal numbers of the exons intended to be analysed should be indicated in the appropriate text box (Figure 3c).

In the output display of the aligned sequences, coloured features are used to annotate the mutations and exon boundaries, facilitating data interpretation. Placing the mouse pointer over an annotated mutation produces a tooltip with information about the amino acid residues involved and the sequence position where the mutation occurs.

**Figure 5:**
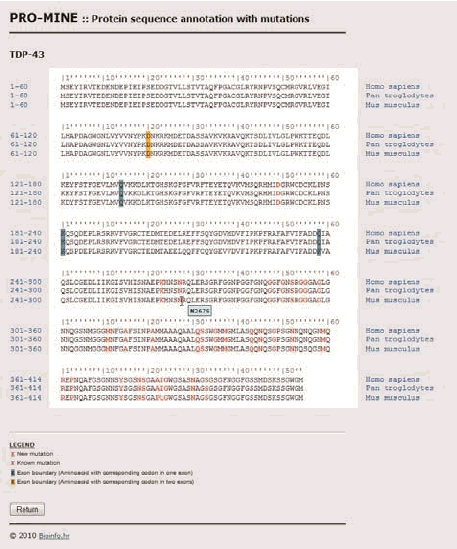
PRO-MINE output: protein sequence annotation with mutations. The aligned TDP-43 protein sequences belonging to *Homo sapiens, Pan troglodytes* and *Mus musculus* are displayed. Marked in orange colour characters are the amino acids involved in the TDP-43 mutations core set. Amino acids located in exons boundaries are highlighted in blue or yellow, depending on whether the corresponding codon is part of one or two exons, respectively. The rectangular tooltip is produced when the mouse is over a certain mutation and displays, in order of appearance: the wild-type amino acid, the position in the protein sequence where the mutation occurs and the mutant amino acid involved.

The described functionality offers a layout for a clear visualisation and analysis of protein sequence changes among organisms in the positions where mutations occur and the distribution of the mutations along the protein sequence. The alignment of the TDP-43 protein sequences from 3 species (*Homo sapiens, Pan troglodytes* and *Mus musculus*) with the TDP-43 mutations core set annotated is shown as an example in Figure 5.

### Future Work

We plan to continue the development of PRO-MINE by extending it to other proteins and by improving its functionalities.

Like TDP-43, mutations in many other genes have been found to be important in FTLD/ALS and have gained the interest from the researchers, such as those identified recently in FUS gene (MIM# 137070) (Lagier-Tourenne and Cleveland, 2009), and in the previously identified *PGRN* (MIM# 138945) (Gijselinck et al., 2008) and *SOD1* genes (MIM# 147450) (Cleveland and Rothstein, 2001). Future work will include expanding the database to cover these genes and eventually other genes encoding disease classes of proteins and their disease-associated mutations. The database will be updated on a regular basis with newly available data.

Incorporating links into the results output to supplementary biological sources of relevance in the research of mutations is part of the ongoing work. We also plan, having in view the flexibility enhancement of PRO-MINE, to provide the possibility to submit not only mutations but also protein sequences as queries.

Finally, it will also be possible in future updates to expand the list of prediction tools available in PRO-MINE as soon as they become publicly available.

## CONCLUSIONS

PRO-MINE is a high-quality manually curated database of *TARDBP* gene mutations and a user-friendly web tool for the automated analysis of known and novel mutations.

The developed web tool simplifies and enhances the time-consuming task of assessing the potential effects of mutations on protein function by providing simultaneous retrieval and display of predictions from separate web tools for a set of submitted mutations. Moreover, up-to-date information as well as visualisation tools are available to complement and improve data evaluation.

PRO-MINE is presented here as a proof of concept for derived databases that combine curated datasets with analytical tools in a single resource. Therefore, in future installations it is intended to broaden the use of the web server by expanding the database to other genes involved in neurodegeneration.

We expect PRO-MINE to become a valuable tool in the study of disease-associated mutations as there is a notable and current increasing interest in the mutation research.
